# Medical Appointments in Berlin

**Published:** 1859-09

**Authors:** 


MEDICAL APPOINTMENTS IN BERLIN.—M. Frerichs, of Breslau, has been appointed to the Chair of Clinical Medicine, which has been vacated by M. Schönlein, after having filled it with distinguished ability for twenty years. M. Frerichs is well known by his researches on Bright's disease, and by his able work on the diseases of the liver, but half of which has appeared.

